# Neoadjuvant chemotherapy-enabled tumor conversion and surgical resection in pediatric primary pulmonary Ewing sarcoma: a case report

**DOI:** 10.3389/fonc.2026.1723340

**Published:** 2026-02-16

**Authors:** Jun-Ping Lin, Chong-Rui Li, Bin Li, Jian-Bao Yang

**Affiliations:** Department of Thoracic Surgery, The Second Hospital & Clinical Medical School, Lanzhou University, Lanzhou, China

**Keywords:** case report, Ewing sarcoma, lobectomy, neoadjuvant chemotherapy, pediatric oncology, primary pulmonary tumor

## Abstract

**Background:**

Ewing sarcoma (EWS) is a highly aggressive malignant tumor that primarily affects the skeletal system in children and adolescents. Primary pulmonary Ewing sarcoma (PPEWS) is extremely rare, particularly in children, with very few cases reported in the literature.

**Case presentation:**

This report describes a 10-year-old girl who was admitted with an intermittent cough lasting over 7 months. Chest computed tomography (CT) revealed a mass in the right lower lung lobe. Bronchoscopic biopsy demonstrated a highly malignant undifferentiated small round cell tumor, with immunohistochemistry confirming EWS (positive for CD99, NKX2.2, and FLI-1). After 8 cycles of VDC/IE neoadjuvant chemotherapy, neoadjuvant therapy enabled tumor conversion from unresectable to resectable status. Postoperative pathology confirmed extraskeletal EWS/peripheral primitive neuroectodermal tumor (pPNET) following right middle and lower lobectomy, with good postoperative recovery. Following a multidisciplinary team (MDT) consensus, the patient initiated adjuvant chemotherapy utilizing the same VDC/IE regimen. As of the latest follow-up, she has successfully completed six cycles of adjuvant chemotherapy, and no clinical or radiological signs of recurrence have been observed.

**Conclusion:**

This case underscores the extreme rarity of PPEWS in children and the complexities of its diagnosis and treatment. Neoadjuvant chemotherapy can facilitate tumor conversion, while surgery plays a pivotal role in localized cases with incomplete chemotherapeutic response. Multidisciplinary management is essential for optimizing outcomes.

## 1 Introduction

Ewing sarcoma (EWS) is a highly aggressive small round blue cell tumor that typically arises in the diaphysis of long bones in children and adolescents. Its hallmark chromosomal translocation, t(11;22)(q24;q12), results in the EWSR1-FLI1 fusion gene, a key driver of tumorigenesis (1). Although extraskeletal EWS accounts for 15–20% of cases, primary pulmonary involvement is exceedingly rare, especially in children under 10 years of age (2).

Recent systematic reviews have documented fewer than 50 cases of primary pulmonary EWS (PPEWS) worldwide, predominantly in adolescents and young adults, with isolated reports in younger children (3). Clinical presentations are often nonspecific respiratory symptoms, such as cough, dyspnea, or chest pain, which can lead to misdiagnosis as pneumonia, tuberculosis, or other common pulmonary conditions (4). Accurate diagnosis requires histopathology, immunohistochemistry, and molecular genetic analysis (e.g., EWSR1 gene rearrangement) (5).

Given its rarity, no standardized treatment protocol exists for PPEWS. Management generally mirrors that of skeletal EWS, incorporating neoadjuvant chemotherapy, surgical resection, and postoperative chemoradiotherapy (6). Neoadjuvant chemotherapy can convert initially unresectable tumors to resectable ones, with surgical resection providing crucial local control, particularly in pediatric cases (7). Herein, we present a 10-year-old girl with PPEWS who achieved successful surgical resection following tumor conversion enabled by neoadjuvant chemotherapy, offering insights into the management of this rare entity.

## 2 Case presentation

**Figure 1:**
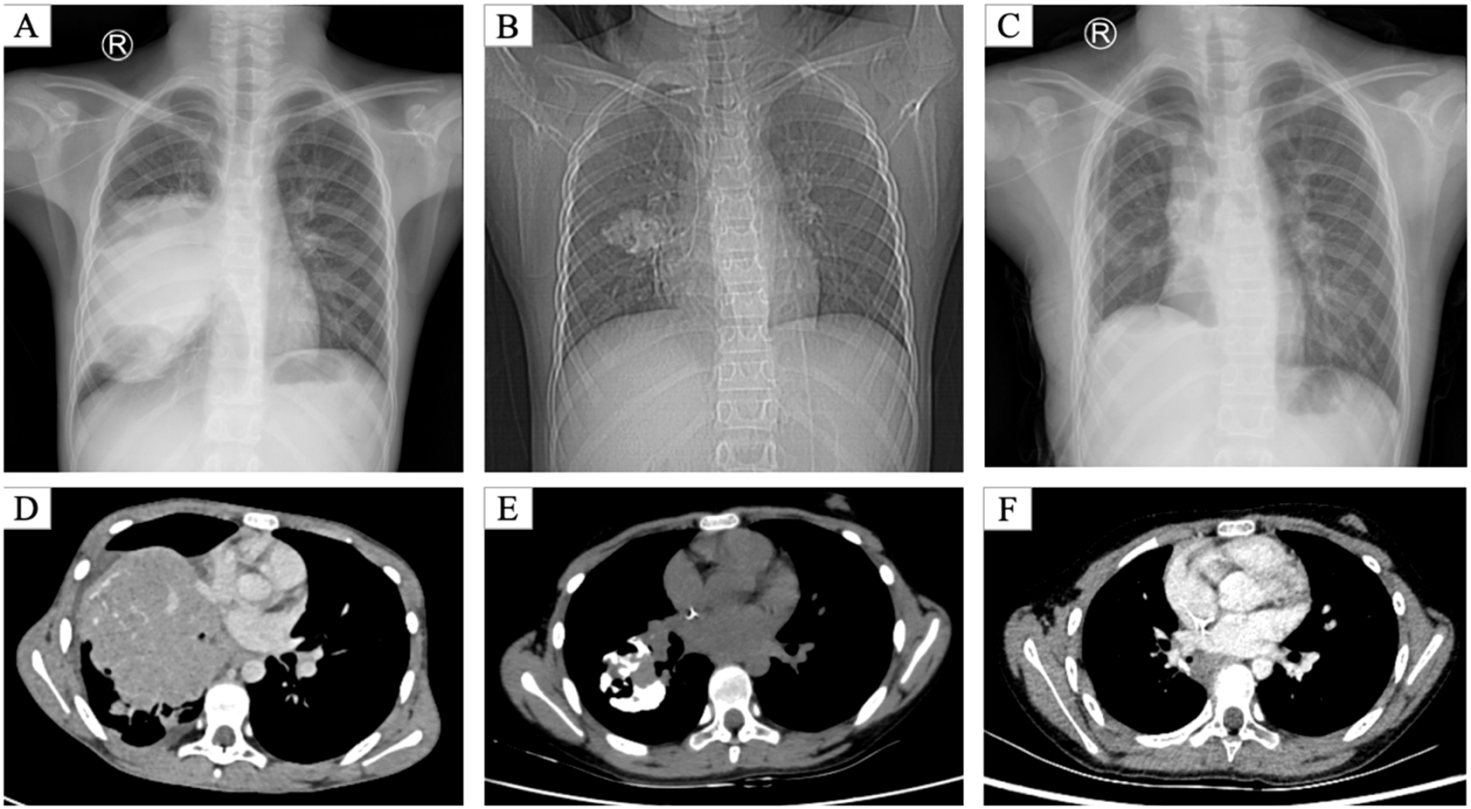
Serial chest radiographs and CT scans. Composite imaging illustrates the tumor’s evolution. **(A)** Pre-chemotherapy posteroanterior chest radiograph showing a large opacity in the right lower lobe with bronchial obstruction, initially misdiagnosed as pneumonia. **(B)** Pre-chemotherapy lateral chest radiograph confirming the mass located posterior to the cardiac silhouette. **(C)** Post-chemotherapy (preoperative) chest radiograph demonstrating partial resolution of the opacity with improved aeration, indicating a partial therapeutic response and conversion to a resectable state. **(D)** Pre-chemotherapy axial CT (lung window) revealing a heterogeneous right lower lobe mass (33 × 32 × 27 mm) with calcification and bronchial compression. **(E)** Post-chemotherapy axial CT showing tumor shrinkage and relief of bronchial obstruction, highlighting the conversion effect. **(F)** One-month postoperative axial CT demonstrating a clear lung field without residual tumor, confirming successful surgical resection. These serial images underscore the role of diagnostic assessment, treatment response, and multidisciplinary management.

We describe the case of a 10-year-old female with a non-contributory medical history who presented with a chronic, intermittent cough of 7 months’ duration. The cough was characterized by significant diurnal variation, peaking during nocturnal and early morning periods, and was conspicuously devoid of expectoration, hemoptysis, or associated dyspnea. At the time of clinical baseline evaluation, anthropometric assessment revealed a height of 145 cm and a weight of 35 kg, with a corresponding body surface area (BSA) of 1.2 m^2^. The absence of systemic constitutional signs, including fever and weight loss, initially confounded the clinical presentation. A chest computed tomography (CT) scan performed at a primary care facility led to a presumptive diagnosis of either right lower lobe pneumonia or an occult endobronchial foreign body. Despite a 48-hour trial of empirical intravenous anti-infective treatment, no symptomatic resolution was achieved. The patient was consequently transferred to our specialized center for intensive diagnostic workup, as her clinical condition remained stagnant (**Figures 1A, D**).

**Figure 2:**
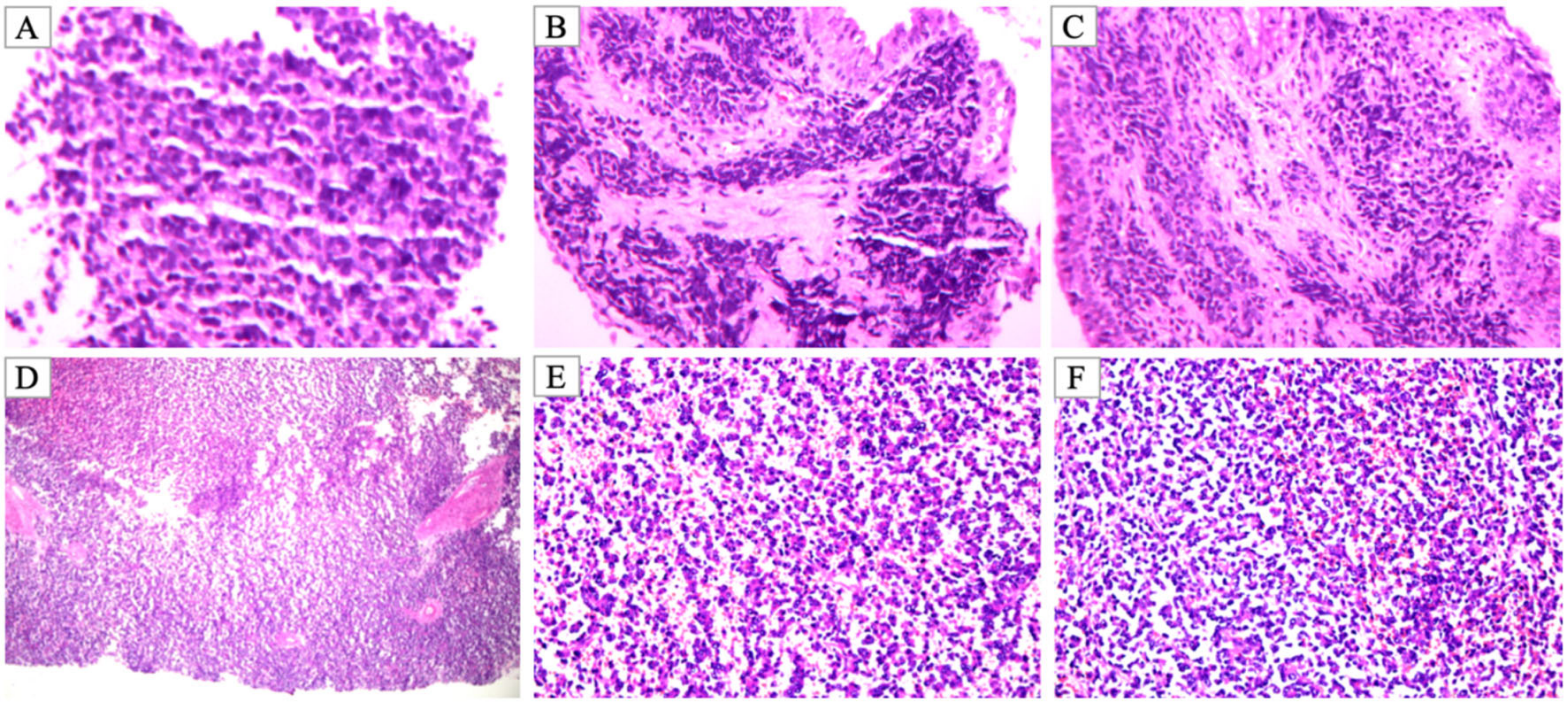
Histopathological findings of pre-biopsy and resected specimens. **(A–C)** Initial endobronchial biopsy showing small round blue cell morphology with scant cytoplasm, arranged in sheets, consistent with a malignant small round cell tumor (H&E staining, magnification ×200). **(D–F)** Postoperative resected specimen demonstrating features of extraskeletal EWS/PNET. Tumor cells are densely packed with hyperchromatic nuclei and inconspicuous nucleoli, displaying a diffuse sheet-like growth pattern [H&E staining, magnification ×100 for D, ×200 for **(E, F)**].

The patient was then referred to our institution for further evaluation. Bronchoscopy identified an obstructing lesion in the right lower lobe bronchus. Biopsy revealed densely packed small round cells with hyperchromatic nuclei and scant cytoplasm. Immunohistochemistry showed positivity for vimentin, CD99, CD56, synaptophysin, and CD117, with a Ki-67 proliferation index of approximately 90%. A highly malignant small round cell tumor was suspected, with differentials including pulmonary blastoma, neuroblastoma, and EWS (**Figures 2A–C**).

**Figure 3:**
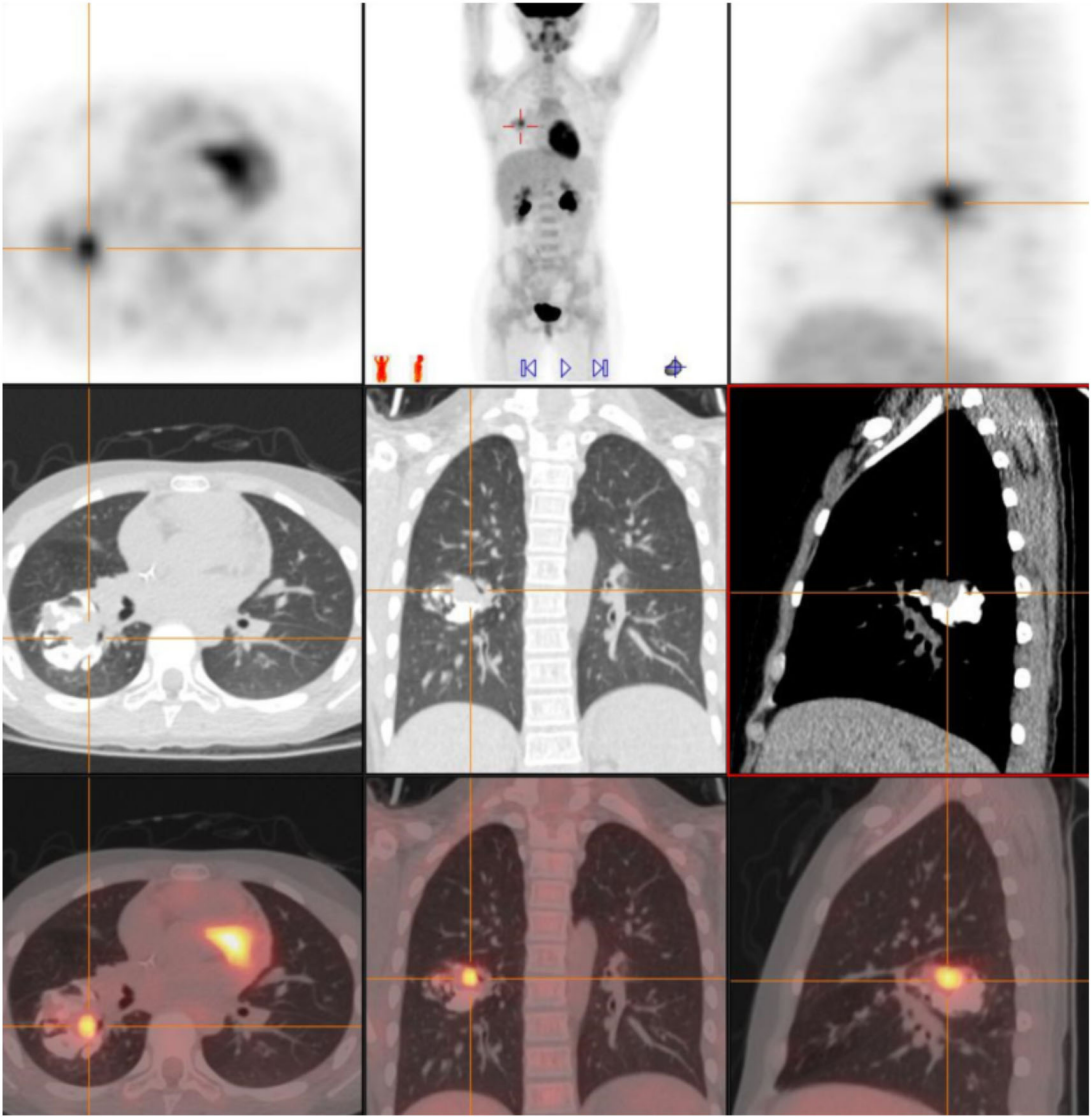
Preoperative PET-CT findings. Preoperative PET-CT demonstrating a heterogeneous mass in the dorsal segment of the right lower lobe (33 × 32 × 27 mm) with coarse calcifications and uneven FDG uptake (SUVmax: 4.9). The lesion caused partial bronchial narrowing, but no abnormal FDG uptake was detected in the mediastinal lymph nodes or distant organs, suggesting localized disease.

Subsequent pathology consultation confirmed PPEWS. Whole-body positron emission tomography-computed tomography (PET-CT) demonstrated a solid mass in the dorsal segment of the right lower lobe, measuring 33 × 32 × 27 mm, with multiple calcifications and heterogeneous FDG uptake (SUVmax: 4.9), but no distant metastases or abnormal lymph nodes. Due to the tumor’s size and location, it was deemed initially unresectable (**Figure 3**).

In December 2024, the patient initiated a neoadjuvant therapeutic protocol consisting of eight alternating cycles of the VDC/IE regimen (vincristine, doxorubicin liposome, and cyclophosphamide alternating with ifosfamide and etoposide) administered at 3-week intervals. To ensure pharmacological precision and optimize the therapeutic index for this pediatric patient, all chemotherapeutic dosages were meticulously titrated based on a calculated body surface area (BSA) of 1.2 m^2^, derived from the baseline height (145 cm) and weight (35 kg) using the Mosteller formula. The specific regimen-defined dose intensities were as follows: (1) VDC cycle: vincristine (1.9 mg, 1.5 mg/m^2^), cyclophosphamide (1.16 g, approx. 1000 mg/m^2^), and doxorubicin liposome (20 mg, approx. 16.7 mg/m^2^) on day 1; (2) IE cycle: ifosfamide (2.2 g/day, 1.8 g/m^2^/day) and etoposide (120 mg/day, 100 mg/m^2^/day) administered from day 1 to day 5.

Post-chemotherapy imaging revealed substantial tumor shrinkage relative to baseline, with residual metabolic activity but alleviated bronchial compression, fulfilling criteria for surgical resection and exemplifying the conversion effect of neoadjuvant therapy (**Figures 1B, E**).

**Figure 4:**
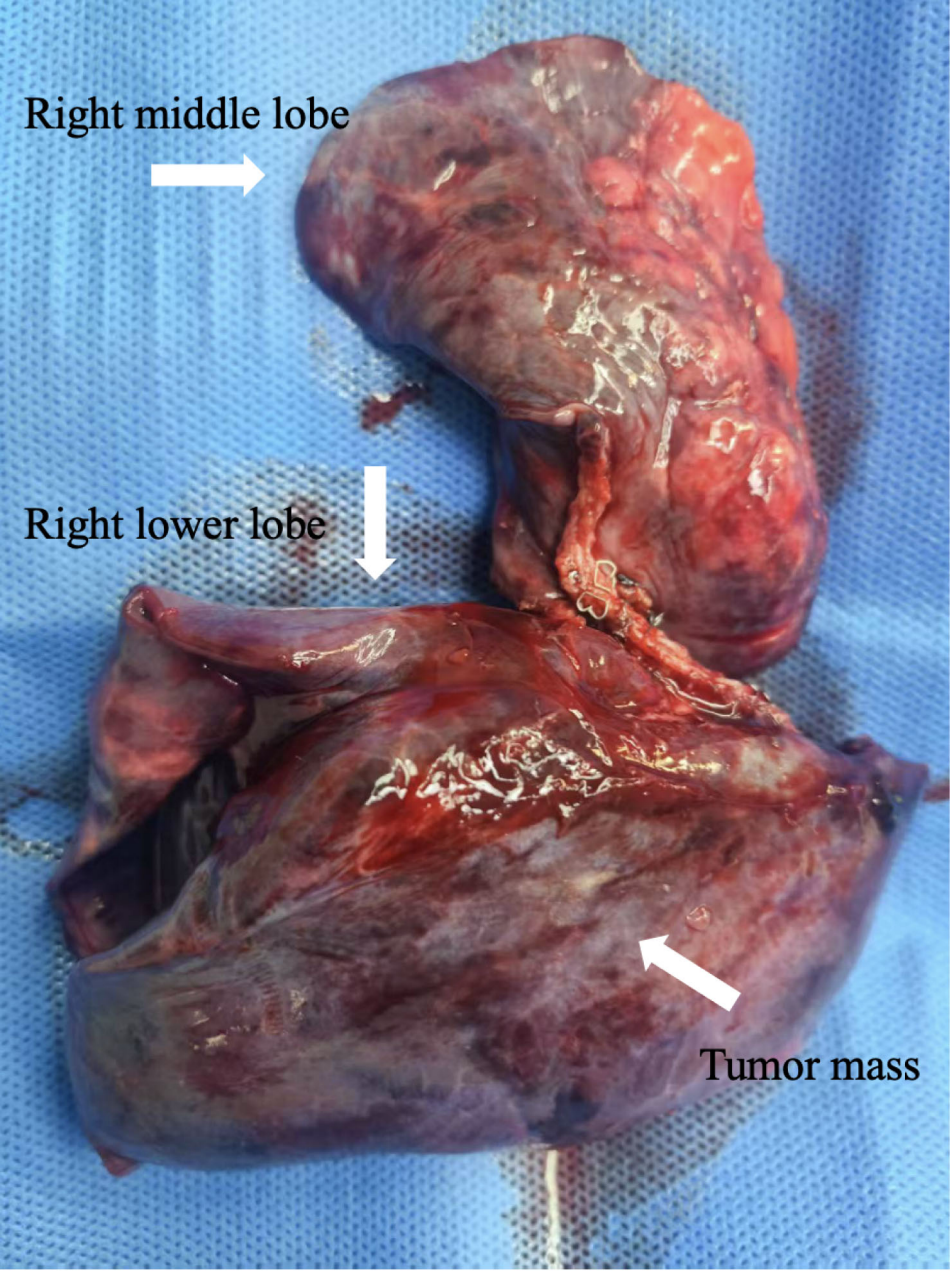
Resected right middle and lower lobes with tumor mass.

Following multidisciplinary team (MDT) discussion, surgery was planned. In May 2025, the patient underwent right middle and lower lobectomy under general anesthesia. Intraoperatively, the tumor exhibited well-defined borders without pleural invasion or mediastinal involvement (**Figure 4**). Postoperative pathology confirmed extraskeletal EWS/pPNET, with immunohistochemistry positive for CD99, vimentin, CD56, NKX2.2, and weakly positive for FLI-1; the Ki-67 index was approximately 60%, suggesting incomplete chemotherapeutic sensitivity. Resection margins were negative, and the 5 examined lymph nodes showed reactive hyperplasia only (**Figures 2D–F**). Postoperative recovery was uncomplicated. Given the high-risk nature of the disease, the MDT recommended eight additional cycles of adjuvant VDC/IE chemotherapy. Currently, the patient has completed six of these adjuvant cycles. She has tolerated the treatment well, and no evidence of recurrence has been detected during the follow-up period. (**Figures 1C, F**).

## 3 Discussion

PPEWS is exceedingly rare in children. EWS primarily arises from bone, with extraskeletal forms comprising only 15–20% of cases; pulmonary primaries are even scarcer, with fewer than 50 reported globally, mainly in adolescents and young adults (8, 9).

Clinical features are nonspecific, often mimicking common conditions like pneumonia or bronchial foreign bodies, thereby risking diagnostic delays. In this case, the insidious presentation with isolated cough evaded diagnosis across multiple facilities, underscoring the need for heightened suspicion in refractory pediatric lung lesions and prompt bronchoscopy with pathological evaluation (10).

Histologically, EWS presents as tightly arranged small round blue cells, with positive immunohistochemical markers such as CD99, NKX2.2, and FLI-1 being of significant diagnostic value. Although molecular tests like FISH or RT-PCR can confirm EWSR1 translocation, in cases with limited specimens or insufficient conditions, combining morphology and immunohistochemistry can yield highly reliable diagnoses (11).

Currently, there is no standard treatment pathway for PPEWS, with comprehensive strategies including neoadjuvant chemotherapy, surgery, and postoperative radiotherapy commonly used. The classic VDC/IE regimen is the first-line recommendation (7). In this case, after completing 8 cycles of neoadjuvant chemotherapy, the tumor significantly shrank, converting the initially unresectable lesion to resectable, highlighting the benefit of conversion therapy. Notably, while the landmark AEWS0031 trial (12) has established the superiority of interval-compressed regimens (VDC/IE every 2 weeks) in enhancing survival outcomes for localized Ewing sarcoma, a traditional 3-week interval was maintained for this patient. This decision was primarily dictated by her critical clinical presentation, characterized by severe endobronchial compression and tenuous respiratory reserve. Given the primary pulmonary origin—a distinct entity from the skeletal cohorts predominantly studied in AEWS0031—the 3-week cycle was prioritized to allow for adequate physiological recovery and to mitigate the risk of acute treatment-related pulmonary toxicity. This individualized approach successfully facilitated significant radiological regression without compromising safety. Although preoperative imaging and postoperative pathology (Ki-67 proliferation index approximately 60%) indicated incomplete chemotherapy response, surgery achieved radical local resection with negative margins and no lymph node metastasis, showing no recurrence on short-term follow-up. The successful administration of six adjuvant chemotherapy cycles without signs of recurrence to date provides preliminary evidence for the feasibility of this intensive systemic consolidation strategy in pediatric PPES. This approach aims to minimize the risk of late relapse, particularly considering the high Ki-67 index identified in the resected specimen. This aligns with reports in the literature on pediatric PPEWS cases, where aggressive surgical intervention can significantly improve prognosis (3, 13). As illustrated in this case, neoadjuvant chemotherapy-enabled tumor conversion paves the way for curative surgical resection, underscoring its pivotal role in localized disease.

Several limitations of this report warrant consideration. First, while the diagnosis of PPES was strongly supported by classical morphology and robust immunohistochemical expression of CD99, NKX2.2, and FLI-1, molecular verification (e.g., FISH or NGS for EWSR1 rearrangement) was not performed due to socio-economic constraints and the clinical urgency of the patient’s respiratory obstruction. Second, owing to institutional policy regarding the digital archiving of histological imagery at the time of diagnosis, high-resolution original micrographs for certain IHC markers, such as CD99 and Ki-67, were not integrated into the hospital’s central imaging system and thus were unavailable for export. While the follow-up period is relatively short, the completion of six adjuvant chemotherapy cycles with no evidence of recurrence underscores the initial clinical stability achieved through this multidisciplinary approach. Continuous long-term monitoring remains essential to evaluate the ultimate oncologic outcome.

From a thoracic surgical viewpoint, this case illustrates surgery’s indispensable role post-conversion, especially in localized PPEWS with suboptimal chemotherapy response, offering curative potential. The process highlights multidisciplinary synergy among pediatric oncology, thoracic surgery, radiology, and pathology. Moreover, it furnishes a practical reference for rare pediatric lung tumors, including precise chemotherapy dosing for analogous scenarios.

## 4 Conclusion

PPEWS represents an extraordinarily rare pediatric malignancy, posing substantial diagnostic and therapeutic hurdles. This case attained resection and favorable short-term prognosis via expeditious pathology and neoadjuvant conversion therapy. It advocates vigilance for rare tumors in persistent pediatric pulmonary lesions and affirms surgery’s centrality in post-conversion localized disease management.

## Data Availability

The original contributions presented in the study are included in the article/supplementary material. Further inquiries can be directed to the corresponding author.
